# Primary Hyperparathyroidism Due to Parathyroid Adenoma in a Post-Kidney Transplant Patient With Lupus Nephritis: A Case Report

**DOI:** 10.7759/cureus.109103

**Published:** 2026-05-18

**Authors:** Hamza Mehmood, Maurice Maglasang, Sepehr Seifi, Charles Wood, Donnie Aultman

**Affiliations:** 1 General Surgery, Edward Via College of Osteopathic Medicine, Monroe, USA; 2 Osteopathic Medicine, New York Institute of Technology College of Osteopathic Medicine at Arkansas State University, Jonesboro, USA; 3 Medicine, Arkansas College of Osteopathic Medicine, Fort Smith, USA; 4 Transplant Surgery, Willis Knighton Health System, Shreveport, USA

**Keywords:** hyperparathyroidism, parathyroid adenoma, parathyroidectomy, post-transplant hypercalcemia, renal transplantation

## Abstract

Hyperparathyroidism is common in patients with existing chronic kidney disease (CKD), typically manifesting as secondary or tertiary hyperparathyroidism. We report a 47-year-old African American woman who initially presented with CKD secondary to lupus nephritis, as well as hyperparathyroidism, thought to be secondary to worsening kidney function. Following a kidney transplant, parathyroid hormone (PTH) was persistently elevated, at levels ranging from 370.4 to 614.9 pg/mL (reference range: 10-65 pg/mL) over the course of 12 months. Uptake on nuclear imaging demonstrated retained radionuclide along the posterior left thyroid lobe, which raised the suspicion for a diagnosis of primary hyperparathyroidism due to a parathyroid adenoma. The need for surgical intervention was supported, with the evidence obtained, and the patient was therefore treated with left inferior parathyroidectomy and has since had no complaints. This case highlights the importance of considering single-gland adenomatous pathology in post-transplant patients with persistent hyperparathyroidism, contrasted with multiple-gland hyperplasia seen in autonomous tertiary hyperparathyroidism, as expected in the usual circumstance.

## Introduction

Hyperparathyroidism is a common complication in patients with existing chronic kidney disease (CKD), typically manifesting as secondary or tertiary hyperparathyroidism [1]. With prolonged CKD, parathyroid gland hyperplasia can become irreversible, resulting in autonomous parathyroid hormone (PTH) secretion despite restoration of renal function via kidney transplantation [2]. The most common cause of tertiary hyperparathyroidism cases is multiglandular hyperplasia [3]. However, cases of single-gland adenomas have rarely been described in the post-transplant setting, mimicking refractory tertiary disease [4]. These cases represent primary hyperparathyroidism, which only becomes clinically evident after normalization of renal function [5]. Distinguishing between tertiary and primary hyperparathyroidism is crucial due to management and treatment being vastly different: subtotal or total parathyroidectomy for tertiary and focused adenoma excision for primary [6,7].

This report describes a 47-year-old African American woman who initially presented with CKD secondary to lupus nephritis as well as hyperparathyroidism, thought to be secondary to worsening kidney function. However, it was later discovered to be due to a single parathyroid adenoma, illustrating the diagnostic challenges when trying to evaluate persistent post-transplant hyperparathyroidism.

## Case presentation

In August 2024, a 47-year-old African American woman with a history of CKD secondary to lupus nephritis, hypertension, hypercalcemia, and hyperparathyroidism presented to Willis-Knighton Medical Center, Shreveport, Louisiana, United States, with complaints of worsening renal function demonstrated by abnormal laboratory results. Initial evaluation revealed that the patient was afebrile with a creatinine of 3.75 mg/dL and an estimated glomerular filtration rate (eGFR) of 15 mL/min/1.73 m², which was calculated using the Chronic Kidney Disease Epidemiology Collaboration (CKD-EPI) equation, with no other complaints. It was determined at this visit that the patient was a good candidate for renal transplantation.

At the time of initial presentation, the patient did not report classic symptoms of hypercalcemia such as bone pain, nephrolithiasis, or gastrointestinal complaints, although hypercalcemia was noted on laboratory evaluation. Preoperative laboratory assessment (Table 1) demonstrated hypercalcemia with markedly elevated PTH (laboratory reference range: 10-65 pg/mL), consistent with either secondary or tertiary hyperparathyroidism. PTH reference intervals are generally age-dependent, but not significantly sex-dependent in adults. Phosphorus and 25-hydroxy vitamin D levels were within normal limits. At this stage, the etiology of hyperparathyroidism remained uncertain, as the biochemical profile was consistent with either secondary hyperparathyroidism due to CKD or a coexisting primary process that was masked by renal dysfunction. In August 2024, the patient underwent a successful transplant procedure, with renal parameters steadily improving (Table 1).

**Table 1:** Serial laboratory values at key clinical time points Summary of relevant laboratory parameters measured pre-transplant, seven months post-transplant, intraoperatively during parathyroidectomy, and three months post-parathyroidectomy. Arrow (->) indicates change from pre-excision to five minutes post-excision. †Intraoperative rapid PTH measurement. Dashes indicate values not measured or not applicable at that time point. Asterisk (*) denotes value outside the laboratory reference range. PTH: parathyroid hormone, eGFR: estimated glomerular filtration rate

Parameter	Pre-transplant	7 months post-transplant	Intraoperative	3 months post-parathyroidectomy	Reference range
Serum calcium (mg/dL)	11.1*	12.7*	-	9.3	8.5-10.5
Intact PTH (pg/mL)	735.1*	462.8*	344.3 → 129.6†*	42	10-65
Phosphorus (mg/dL)	2.8	3.5	-	3.3	2.5-4.5
25-Hydroxy vitamin D (ng/mL)	49.3	-	-	53.2	30-100
Creatinine (mg/dL)	3.75*	1.65*	-	1.26	0.7-1.3
eGFR (mL/min/1.73 m²)	15*	38.3*	-	44.1*	≥60

**Figure 1:**
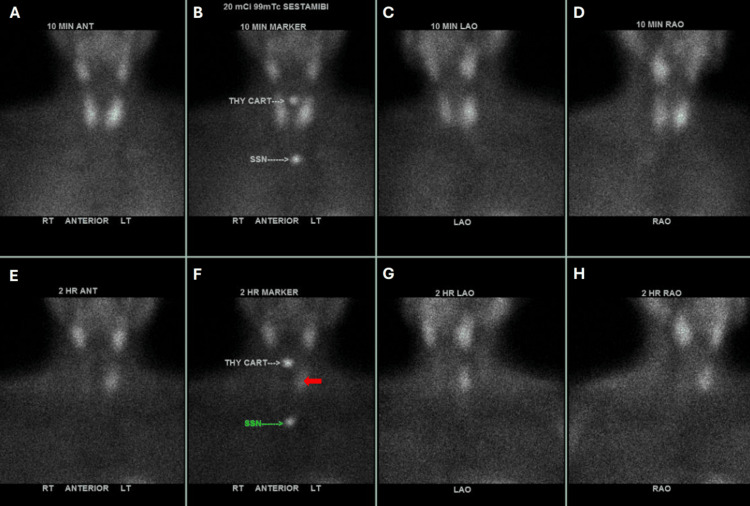
Technetium-99m (99mTc) sestamibi parathyroid scintigraphy (A-D) Early 10-minute planar images. (E-H) Delayed two-hour planar images demonstrating near-complete thyroid washout with persistent focal radionuclide retention (red arrow) posterior to the left thyroid lobe, most prominent in panel F, consistent with a left parathyroid adenoma. White arrows indicate anatomic landmarks. THY CART: thyroid cartilage, SSN: suprasternal notch, RT: right, LT: left, ANT: anterior, LAO: left anterior oblique, RAO: right anterior oblique

Seven months post-transplant, in March 2025, the patient developed symptoms consistent with hypercalcemia, including bowel disturbances and hair loss. Laboratory evaluation (Table 1) revealed persistent hypercalcemia with inappropriately elevated intact PTH. Given the persistence of apparent hyperparathyroidism despite restored renal function, a nuclear medicine scan of the thyroid and parathyroid was indicated. The scan demonstrated a normal thyroid with retained radionuclide along the posterior aspect of the left thyroid lobe, consistent with a left-sided parathyroid adenoma (Figure 1).

**Figure 2:**
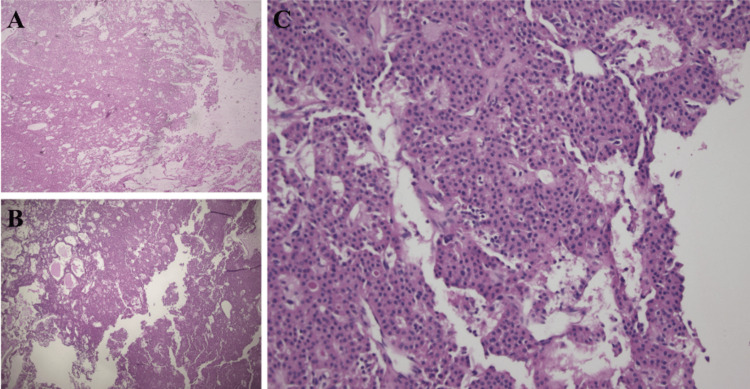
Histopathology of the excised left inferior parathyroid gland (A) Frozen section at 40× showing hypercellular parathyroid tissue with reduced adipose stroma. (B) Permanent H&E section at 40× confirming diffuse hypercellularity. (C) Permanent H&E section at 200× demonstrating sheets of chief cells. The gland measured 3.1 × 1.8 × 1.0 cm and weighed 2,585 mg. H&E: hematoxylin and eosin

In August 2025, the patient underwent a focused left inferior parathyroidectomy for suspected parathyroid adenoma. An incision was made in the lower neck through the skin and platysma, and a TruNode probe estimated very high uptake of over 1,800 counts per second in the left lower neck. Further dissection at this site revealed a large parathyroid gland weighing 2,585 mg, which was confirmed via frozen section (Figure 2). Intraoperative PTH monitoring demonstrated a decline from 344.3 pg/mL to 129.6 pg/mL within 5 minutes of gland removal, confirming successful adenoma excision. The patient was discharged in stable condition with close outpatient observation and follow-up. At three-month post-parathyroidectomy follow-up, calcium and PTH had normalized (Table 1).

## Discussion

Persistent hyperparathyroidism following kidney transplantation is a well-recognized consequence of long-standing parathyroid hyperplasia that fails to regress despite restoration of renal function [1,2]. Recent multicenter studies have shown that persistent hyperparathyroidism occurs in approximately 62% of kidney transplant patients during the first year. Of these, about 21% meet the criteria for tertiary hyperparathyroidism, defined by concomitant hypercalcemia and elevated PTH [8]. Elevated pre-transplant PTH (≥300 pg/mL) and prior calcimimetic exposure were identified as significant predictors of persistent and tertiary hyperparathyroidism [8,9]. Morphologically, tertiary disease most often represents multinodular hyperplasia, as shown by Krause and Hedinger, who reported hyperplasia in 85% of 41 patients and solitary adenoma in only 5% [10]. Nevertheless, single-gland adenomas have been described in the post-transplant setting and may mimic refractory tertiary disease [4,11]. This distinction carries critical therapeutic implications, as tertiary hyperparathyroidism generally requires subtotal or total parathyroidectomy, whereas adenomatous disease is treated with focused excision [5,6,12]. The present case underscores the diagnostic challenge and highlights the need for vigilance when evaluating persistent hypercalcemia with elevated PTH after transplantation.

Persistent elevation of PTH after kidney transplantation often reflects long-standing changes in parathyroid glands that make them less responsive to normal calcium feedback. Over time, chronic stimulation from kidney disease causes the glands to grow and transition from diffuse to nodular hyperplasia, with some nodules becoming fully independent [13-15]. These nodular areas lose sensitivity to calcium and vitamin D because of the decreased expression of their respective receptors, which allows continued PTH secretion even after renal function has been restored. Messa et al. described this as a shift in the calcium-PTH “set point,” meaning that higher calcium levels are required to suppress hormone release [13]. Similar findings have been shown histologically, where nodular tissue tends to persist after transplantation while the diffuse portions regress [14]. Other studies have linked this persistence to factors such as extended dialysis duration, elevated calcium-phosphate product, and prior calcimimetic use, each of which can lead to irreversible structural and functional changes in the glands [16]. Interestingly, persistent hyperparathyroidism has also been reported in patients who never needed dialysis treatment, which suggests that gland autonomy may start even before end-stage kidney disease and only becomes obvious once kidney function normalizes [17]. This patient showed a similar pattern of persistent PTH elevation despite improvement in renal function. However, unlike diffuse multiglandular involvement that is typical of tertiary hyperparathyroidism, imaging revealed radionuclide uptake that was localized in a single gland. This finding, combined with the significant drop in PTH after excision and complete normalization of PTH at three-month follow-up, supports the presence of a solitary adenoma rather than widespread hyperplasia. This case underscores how chronic overstimulation may blur the line between tertiary and primary disease. As described by Tominaga et al., long-standing secondary hyperplasia may gradually evolve toward a single adenomatous focus with autonomous hormone secretion [18].

The laboratory results in this patient were not sufficient to clearly differentiate between tertiary and primary hyperparathyroidism, as calcium and PTH levels can overlap significantly in transplant recipients [3,7]. Given the inconclusive biochemical findings, imaging studies were performed to help guide surgical planning. Although sestamibi scans may have limitations when dealing with multinodular renal hyperparathyroidism, focal radionuclide uptake is more consistent with single-gland disease [10]. While rare in kidney transplant recipients, solitary parathyroid adenomas have been reported as a cause of ongoing hyperparathyroidism in these patients [4]. In this instance, focal uptake observed posterior to the left thyroid lobe supported a diagnosis of a single adenoma and warranted a focused surgical intervention. Following removal of the gland, PTH levels decreased appropriately, confirming that the primary source of excess hormone production had been eliminated.

Clinically, this distinction is important because management strategies differ significantly depending on the underlying pathology. While tertiary hyperparathyroidism typically requires subtotal or total parathyroidectomy due to multiglandular involvement, solitary adenomatous disease can be effectively treated with focused excision. Therefore, persistent hypercalcemia with elevated PTH after kidney transplantation should not automatically be attributed to tertiary hyperparathyroidism. Careful correlation of biochemical findings with imaging and intraoperative PTH response is essential to identify patients with single-gland disease and ensure appropriate surgical management.

## Conclusions

This case reinforces that persistent hyperparathyroidism after kidney transplantation should not automatically be assumed to represent tertiary disease. When renal function has recovered, but hypercalcemia and elevated PTH levels persist, the possibility of a single adenoma should be considered. Careful localization and intraoperative hormone monitoring allow for focused parathyroidectomy, resulting in reliable biochemical correction.
